# Supplementary material for: The effectiveness of putative wearable repellent technologies to protect against mosquito biting and Aedes-borne diseases, and their economic impact
Source: PLoS Negl Trop Dis. 2024 Dec 18;18(12):e0012621. doi: 10.1371/journal.pntd.0012621 (PMC11694967; doi:10.1371/journal.pntd.0012621)

**Supplementary Figure 1. Epidemic curves for arbovirus outbreaks with different R_0_ values and no interventions (no repellent use). Parameters as in Table 2. Final proportion of humans infected at end of outbreak: ZKV, R_0_=2.2 – 98.7%; ZKV, R_0_=1.6 – 92.6%; DNV, R_0_=2.6 – 99.1%; DNV, R_0_=1.4 – 76.6%, CHKV, R_0_=2.2 – 99%, CHKV, R_0_=1.4 – 78.2%.**


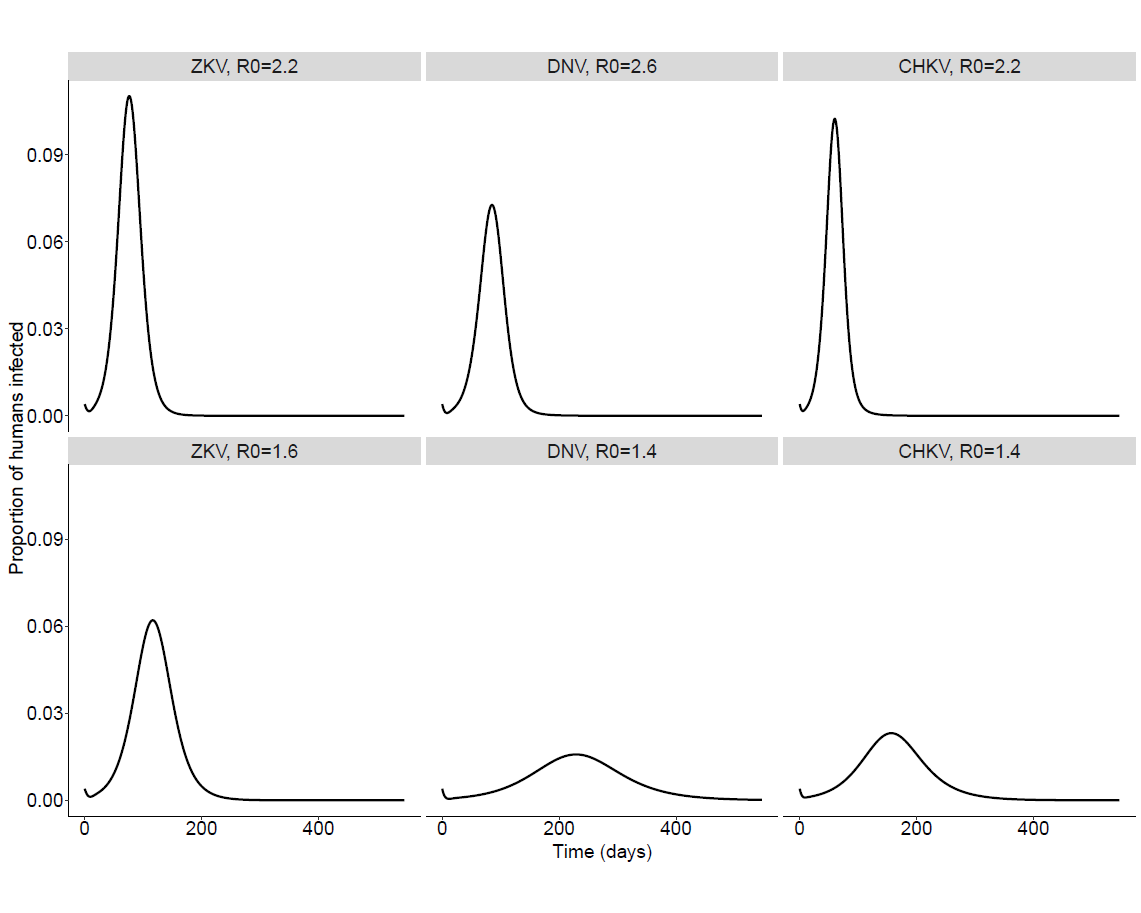

Supplement: S1 Fig — Parameters as in Table 2. Final proportion of humans infected at end of outbreak: ZKV, R0 = 2.2–98.7%; ZKV, R0 = 1.6–92.6%; DNV, R0 = 2.6–99.1%; DNV, R0 = 1.4–76.6%, CHKV, R0 = 2.2–99%, CHKV, R0 = 1.4–78.2%. (DOCX) [file pntd.0012621.s001.docx]
