# Supplementary material for: The effectiveness of putative wearable repellent technologies to protect against mosquito biting and Aedes-borne diseases, and their economic impact
Source: PLoS Negl Trop Dis. 2024 Dec 18;18(12):e0012621. doi: 10.1371/journal.pntd.0012621 (PMC11694967; doi:10.1371/journal.pntd.0012621)

**Supplementary Figure 2. Mean protection (±S.E. bars) provided by 20% DEET topically applied to the distal part of the lower arm. The proximal part of the lower arm was covered with untreated cotton fabric. n = 3 replicates. Protective efficacy was determined as a proportion of the number of mosquito probings on the treated arm in relation to the number of probings on the control arm.**


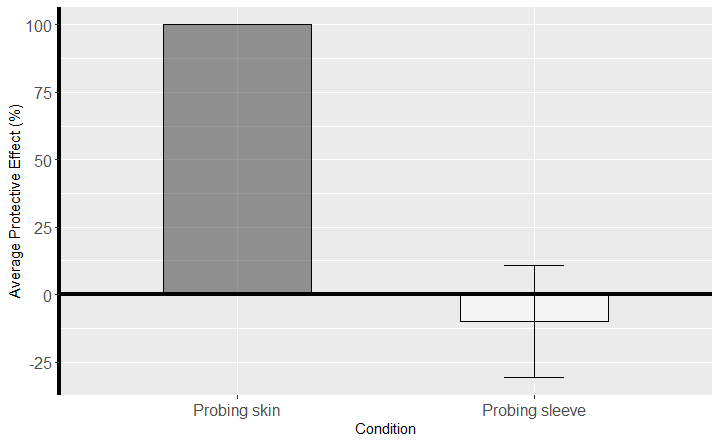

Supplement: S2 Fig — The proximal part of the lower arm was covered with untreated cotton fabric. n = 3 replicates. Protective efficacy was determined as a proportion of the number of mosquito probings on the treated arm in relation to the number of probings on the control arm. (DOCX) [file pntd.0012621.s002.docx]
