# Supplementary material for: The effectiveness of putative wearable repellent technologies to protect against mosquito biting and Aedes-borne diseases, and their economic impact
Source: PLoS Negl Trop Dis. 2024 Dec 18;18(12):e0012621. doi: 10.1371/journal.pntd.0012621 (PMC11694967; doi:10.1371/journal.pntd.0012621)

**Supplementary Figure 3. Mean protection (±S.E. bars) provided by three commercially-available laundry additive repellents. Efficacy was determined by arm-in-cage tests using treated fabric that covered the whole of the lower arm. MosquitNo contained the repellent IR3535®, and NoMo and Si Repel contained citronella oil. n = 5 replicates.**


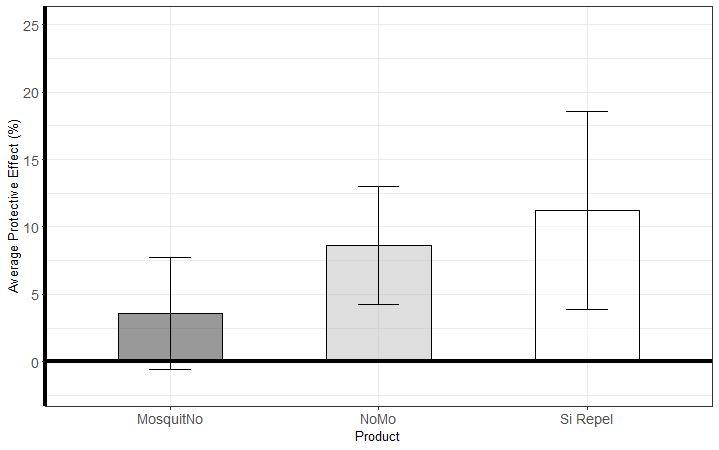

Supplement: S3 Fig — Efficacy was determined by arm-in-cage tests using treated fabric that covered the whole of the lower arm. MosquitNo contained the repellent IR3535, and NoMo and Si Repel contained citronella oil. n = 5 replicates. (DOCX) [file pntd.0012621.s003.docx]
