# Supplementary material for: The effectiveness of putative wearable repellent technologies to protect against mosquito biting and Aedes-borne diseases, and their economic impact
Source: PLoS Negl Trop Dis. 2024 Dec 18;18(12):e0012621. doi: 10.1371/journal.pntd.0012621 (PMC11694967; doi:10.1371/journal.pntd.0012621)

**Supplementary Figure 4. Heatmap for all arbovirus outbreak scenarios. No difference in infectiousness between symptomatic and asymptomatic individuals.**


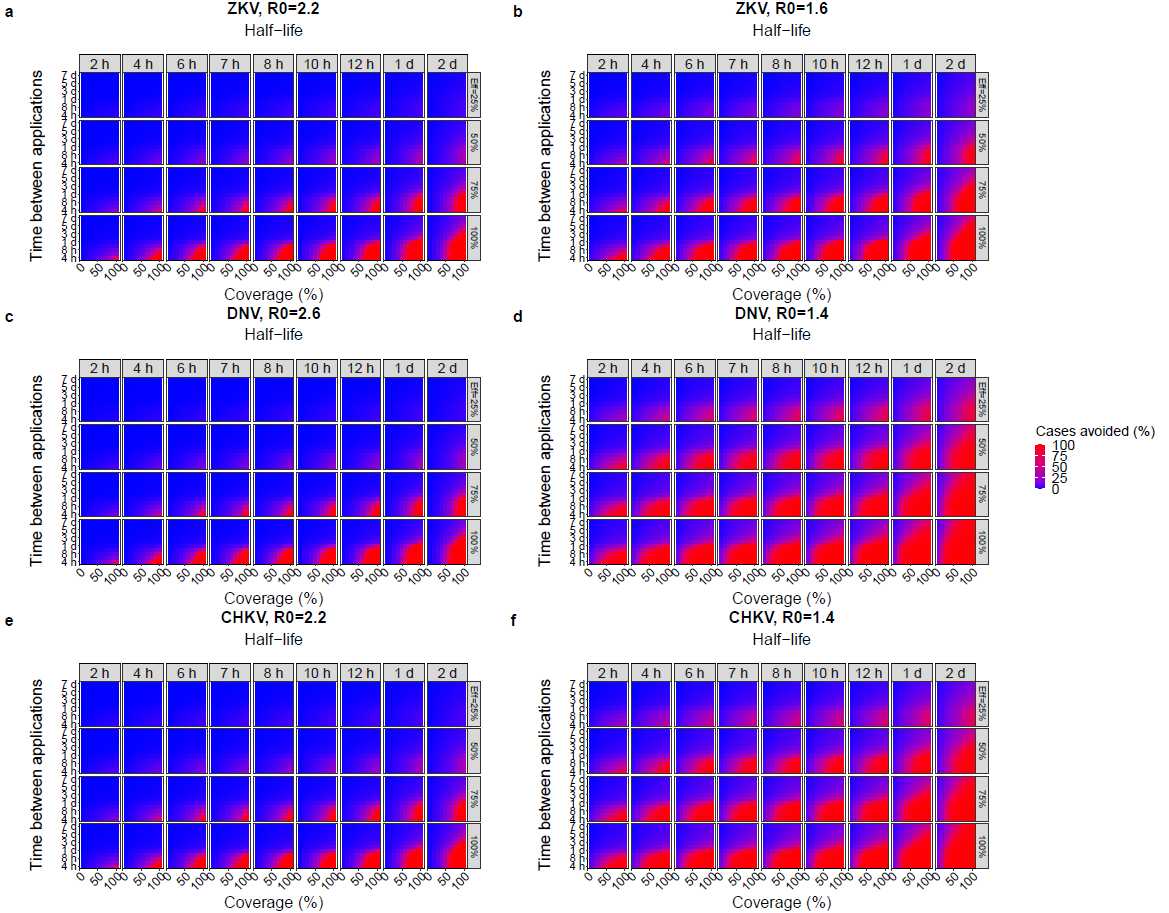

Supplement: S4 Fig — No difference in infectiousness between symptomatic and asymptomatic individuals. (DOCX) [file pntd.0012621.s004.docx]
