# Supplementary material for: The effectiveness of putative wearable repellent technologies to protect against mosquito biting and Aedes-borne diseases, and their economic impact
Source: PLoS Negl Trop Dis. 2024 Dec 18;18(12):e0012621. doi: 10.1371/journal.pntd.0012621 (PMC11694967; doi:10.1371/journal.pntd.0012621)
Supplement: S1 Table — *Based on 5.52 million pregnancies per year in Brazil (population 214.3 million). Equations for cost calculations. Equations were customised depending on available data. Where possible cost per case (mild, severe, etc.) was used which already considers average duration of disease. (DOCX) [file pntd.0012621.s006.docx]

**Supplementary Table 1. Assumptions used in calculations of healthcare costs and cost of lost labour avoided by repellent use in 2023 US dollars. *Based on 5.52 million pregnancies per year in Brazil (population 214.3 million)** ^1^**.**

| Cost measure | CHKV | DNV | ZKV |
| --- | --- | --- | --- |
| % symptomatic (any severity) | 0.83 ^2^ | 0.45 (for primary infections) ^3^ | 0.5 ^4^ |
| % severe disease | 2% (reported 0.6-13%) ^5^ | 6% ^6^ | 0.04% (reported 0.02-0.08%) GBS ^7^  1% (reported 0.49-2.1%) ^7^ |
| % symptomatic patients developing chronic disease | 50% (40-80%) ^5^ | NA | NA |
| % population pregnant | NA | NA | 2.6% * |
| Hospitalisation cost | $2,923 ^8^ | $1,234 ^9^ | $35,555 GBS ^7^  $112,399 Microcephaly ^7^ |
| Outpatient cost | $96.13 acute phase ^10^  $2,510 chronic phase ^10^ | $126 ^9^ | $2.55 ^11^ |
| Value of lost labour | $881 (over the first two months after infection) ^12^  $339 (additional cost in 88% of those with chronic disease up to 6 months after infection) ^12^  $183 (additional cost in 9% of those with chronic disease up to 12 months after infection) | $199 mild disease ^9^  $444 severe disease ^9^ | $25.16 mild disease ^11^  $9,225 GBS ^11^ |

**Equation for *R_0_* for an arboviral disease given the model in Table 1 (main text)**

$$R_{0}=\sqrt{\frac{\beta_{H}\beta_{v}a_{v}}{\gamma\delta(\delta+a_{v})}}$$

$\beta_{H}$: Transmission rate from mosquitoes to humans, $\beta_{v}:$ Transmission rate from humans to mosquitoes, $a_{v}:$ Rate at which infected mosquitoes become infectious, $\delta$: Mosquito death/birth rate per day, $\gamma$: Rate at which infected humans recover.

**Equations for cost calculations**

Equations were customised depending on available data. Where possible cost per case (mild, severe, etc) was used which already considers average duration of disease.

Average healthcare costs per person from Zika virus infection averted by repellent use:

$${cost}_{HC,ZKV}=(p_{symptom, ZKV}{cost}_{mild,ZKV}+p_{GBS}{cost}_{GBS}+p_{pregnant}p_{microcephaly}{cost}_{microcephaly})p_{infections averted,ZKV}$$

Average cost of lost labour per person from ZKV infection averted by repellent use:

$${cost}_{lost labor,ZKV}=(p_{symptom,ZKV}{cost}_{lost labour mild,ZKV}+p_{GBS}{cost}_{lost labour GBS})p_{infections averted,ZKV}$$

Average healthcare costs per person from dengue virus infection averted by repellent use:

$${cost}_{HC,DNV}=(p_{symptom,DNV}{(1-p_{severe,DNV})cost}_{mild,DNV}+p_{symptom,DNV}p_{severe,DNV}{cost}_{severe,DNV})p_{infections averted,ZKV}$$

Average cost of lost labour per person from DNV infection averted by repellent use:

$${cost}_{lost labour,DNV}=(p_{symptom,DNV}{(1-p_{severe,DNV})cost}_{lost labour mild,DNV}+p_{symptom,DNV}p_{severe,DNV}{cost}_{lost loabour severe,DNV})p_{infections averted,DNV}$$

Average healthcare costs per person from chikungunya virus infection averted by repellent use:

$${cost}_{HC,CHKV}=(p_{symptom,CHKV}{\left( 1-p_{severe,CHKV} \right)cost}_{mild,CHKV}+p_{symptom,CHKV}p_{severe,CHKV}{cost}_{severe,CHKV}+ p_{symptom,CHKV}p_{chronic,CHKV}{cost}_{chronic,CHKV})p_{infections averted,CHKV}$$

Average cost of lost labour per person from CHKV infection averted by repellent use:

$${cost}_{lost labour, CHKV}=(p_{symptom,CHKV}{cost}_{lost labour acute,CHKV}+ p_{symptom}p_{chronic,CHKV}p_{sick 6months,CHKV}{cost}_{lost labour 6months,CHKV}+p_{symptom}p_{chronic,CHKV}p_{sick 12months,CHKV}{cost}_{lost labour 12months,CHKV})p_{infections averted,CHKV}$$

| ${cost}_{HC,ZKV}$ | Healthcare costs incurred from symptomatic ZKV infection |
| --- | --- |
| ${cost}_{HC,DNV}$ | Healthcare costs incurred from symptomatic DNV infection |
| ${cost}_{HC,CHKV}$ | Healthcare costs incurred from symptomatic CHKV infection |
| $p_{symptom, ZKV}$ | Probability of symptomatic ZKV infection |
| $p_{symptom, DNV}$ | Probability of symptomatic DNV infection |
| $p_{symptom, CHKV}$ | Probability of symptomatic CHKV infection |
| $p_{infections averted,ZKV}$ | Proportion of ZKV infections averted |
| $p_{infections averted,DNV}$ | Proportion of DNV infections averted |
| $p_{infections averted,CHKV}$ | Proportion of CHKV infections averted |
| $p_{GBS}$ | Probability of developing Guillain-Barré Syndrome following ZKV infection |
| $p_{pregnant}$ | Proportion of population pregnant |
| $p_{microcephaly}$ | Probability of a pregnant woman giving birth to a child with microcephaly |
| $p_{severe,DNV}$ | Probability of severe disease following infection with DNV |
| $p_{severe,CHKV}$ | Probability of severe disease following infection with CHKV |
| $p_{chronic,CHKV}$ | Probability of developing chronic disease following symptomatic CHKV infection |
| $p_{sick 6months,CHKV}$ | Probability of workplace absenteeism due to chronic CHKV disease 6 months after infection |
| $p_{sick 12months,CHKV}$ | Probability of workplace absenteeism due to chronic CHKV disease 12 months after infection |
| ${cost}_{mild,ZKV}$ | Average healthcare costs per mild ZKV case |
| ${cost}_{mild,DNV}$ | Average healthcare costs per mild DNV case |
| ${cost}_{mild,CHKV}$ | Average healthcare costs per mild CHKV case |
| ${cost}_{GBS}$ | Average healthcare costs per case of Guillain-Barré Syndrome caused by ZKV infection |
| ${cost}_{microcephaly}$ | Average healthcare costs per microcephaly case caused by ZKV infection |
| ${cost}_{severe,DNV}$ | Average healthcare costs per severe DNV case |
| ${cost}_{severe,CHKV}$ | Average healthcare costs per severe CHKV case |
| ${cost}_{chronic,CHKV}$ | Average healthcare costs per chronic CHKV case |
| ${cost}_{lost labour mild,ZKV}$ | Average cost of lost labour per mild ZKV case |
| ${cost}_{lost labour mild,DNV}$ | Average cost of lost labour per mild DNV case |
| ${cost}_{lost labour mild,CHKV}$ | Average cost of lost labour per mild CHKV case |
| ${cost}_{lost labour GBS}$ | Average cost of lost labour per Guillain-Barré Syndrome case caused by ZKV infection |
| ${cost}_{lost loabour severe,DNV}$ | Average cost of lost labour per severe DNV case |
| ${cost}_{lost labour acute,CHKV}$ | Average cost of lost labour per acute CHKV case |
| ${cost}_{lost labour 6months,CHKV}$ | Average cost of lost labour of a chronic CHKV case at 6 months following infection |
| ${cost}_{lost labour 12months,CHKV}$ | Average cost of lost labour of a chronic CHKV case at 12 months following infection |

**References**

1. Guttmacher Institute. Country profile Brazil: Unintended pregnancy and abortion. https://www.guttmacher.org/regions/latin-america-caribbean/brazil (2023).

2. Yakob, L. & Clements, A. C. A. A Mathematical Model of Chikungunya Dynamics and Control: The Major Epidemic on Réunion Island. *PLoS One* **8**, e57448 (2013).

3. Ferguson, N. M. *et al.* Benefits and risks of the Sanofi-Pasteur dengue vaccine: Modeling optimal deployment. *Science* **353**, 1033–1036 (2016).

4. Mitchell, P. K. *et al.* Reassessing Serosurvey-Based Estimates of the Symptomatic Proportion of Zika Virus Infections. *Am J Epidemiol* **188**, 206 (2019).

5. Bartholomeeusen, K. *et al.* Chikungunya fever. *Nature Reviews Disease Primers 2023 9:1* **9**, 1–21 (2023).

6. Moraes, G. H., Duarte, E. D. F. & Duarte, E. C. Determinants of Mortality from Severe Dengue in Brazil: A Population-Based Case-Control Study. *Am J Trop Med Hyg* **88**, 670–676 (2013).

7. Alfaro-Murillo, J. A. *et al.* A Cost-Effectiveness Tool for Informing Policies on Zika Virus Control. *PLoS Negl Trop Dis* **10**, e0004743 (2016).

8. de Margarette Oliveira de Andrade, M. *et al.* Chikungunya in Brazil: An epidemic of high cost for private healthcare, 2017. *Trop Med Int Health* **27**, 925–933 (2022).

9. Shepard, D. S., Undurraga, E. A., Halasa, Y. A. & Stanaway, J. D. The global economic burden of dengue: a systematic analysis. *Lancet Infect Dis* **16**, 935–941 (2016).

10. Cardona-Ospina, J. A., Villamil-Gómez, W. E., Jimenez-Canizales, C. E., Castañeda-Hernández, D. M. & Rodríguez-Morales, A. J. Estimating the burden of disease and the economic cost attributable to chikungunya, Colombia, 2014. *Trans R Soc Trop Med Hyg* **109**, 793–802 (2015).

11. Peixoto, H. M., Romero, G. A. S., De Araújo, W. N. & Fernandes de Oliveira, M. R. Guillain-Barré syndrome associated with Zika virus infection in Brazil: a cost-of-illness study. *Trans R Soc Trop Med Hyg* **113**, 252–258 (2019).

12. Feldstein, L. R. *et al.* Estimating the cost of illness and burden of disease associated with the 2014–2015 chikungunya outbreak in the U.S. Virgin Islands. *PLoS Negl Trop Dis* **13**, e0007563 (2019).
