# Supplementary material for: The effectiveness of putative wearable repellent technologies to protect against mosquito biting and Aedes-borne diseases, and their economic impact
Source: PLoS Negl Trop Dis. 2024 Dec 18;18(12):e0012621. doi: 10.1371/journal.pntd.0012621 (PMC11694967; doi:10.1371/journal.pntd.0012621)
Supplement: Attachment — Submitted filename: Response to reviewers.docx [file pntd.0012621.s007.docx]

London School of Hygiene & Tropical Medicine

Department of Disease Control

Keppel Street

London

WC1E 7HT

United Kingdom

4^th^ October 2024

Dear Dr Beebe,

Thank you for your review of our article. We have carefully considered the reviewer comments, and addressed each in turn. We have indicated below how each comment has been addressed.

We hope that you will find this work now ready for publication.

Yours sincerely,

Dr Robert Jones

**Reviewer's responses to comments**

Reviewer #1: The methods are clearly written and understood.

L25: Can the authors provide further information about how probing mosquitoes were counted? *Aedes aegypti* are known to be skittish and probe the skin for a short amount of time, leave and the return to recommence probing. Are counts the total number of mosquitoes that land on the arm during the 75 second period with the potential for a single mosquito in the cage to be counted more than once?

*This has been clarified.*

L91: Can the authors provide further information on how the repellent was applied to skin. Note that the amount of product was applied at 0.33ul/cm2, however was this applied in a spray, cream or roll on application? This information is important in order for the reader to understand and draw conclusions on whether application technique compliance would be reasonably expected by the public.

*This has been clarified. Although we would not expect members of the public to use a pipette and gloved finger to apply the product, this standard method allows for controlling the dose across replicates.*

L119: Can the authors provide further information on how the cotton fabric was treated with the spray on repellent treatments. The authors state that 1ml of repellent is mixed with 9ml of ethanol. Is the entire 10ml of mixed formulation applied to the fabric? How is it applied? If not all of the 10ml of mixed product was applied, how do the authors ensure consistency of application?

*The full amount of 10 ml was applied using a spray bottle. This detail has been added.*

L94: Can the authors please provide more information on the colony of Aedes aegypti used? How were the mosquitoes reared? Were they starved of sucrose prior to the probing tests? Are they known to carry insecticide resistance mutations? What is the colonies origin and how many generations has it been kept in colony?

*Details have been added. This strain has been used in our previous published work, such as Banks et al., Permethrin-treated clothing as protection against the dengue vector, Aedes aegypti: extent and duration of protection, PLoS NTDs 2015.*

Figure 1: When the arm is covered with cotton fabric, is this fabric skin tight against the skin and secured? Please provide more information on how the arm is covered in fabric.

*Details have been added.*

L90: Can the authors provide information on the human participants? Was there only one human participant for all probing assays or are different personnel used? Was human ethics approval sought for the use of human participants in the probing assays? Please provide further information.

*Details have been provided. LSHTM does not require ethics committee review of studies of this nature involving study staff and not recruited members of the public.*

Reviewer #1: The analysis in this manuscript is adequate for the aims of the paper. The results are clearly presented and all relevant data is available to the reader. Request the authors consider the use of different coloured lines to clearly present results in Figure 4 (suggest black for dUDL and red for PMD for example as used in Figure 7b). Nil other comments regarding presentation of results.

*The figure has been revised.*

Reviewer #1: Conclusions are well supported by the data presented however more discussion is required on the practicality and limitations of the public applying the repellents in order to achieve high protection rates. More information on the application methods in the methods section will provide information to the reader on the level of protection afforded by imperfect application. The authors should also discuss the potential limitations of the study given it was performed with colony-reared Aedes aegypti.

*We have added a brief paragraph to the discussion to reflect these points, but note that the need for consumer education and behaviour change are already discussed in the context of the challenges of achieving the highest level of coverage and protection. Further details on application methods have been provided.*

Reviewer #1: The manuscript "The effectiveness of putative wearable repellent technologies to protect against mosquito biting and Aedes-borne diseases, and their economic impact" is an interesting, well written paper providing useful and interesting information on wearable repellent technologies. The paper is easily understood and the data is well presented. The authors should ensure that the methods are detailed enough so that the reader can understand exactly how repellents were applied. This allows the reader to draw conclusions on the applicability of these results in a public health setting.

*Further details on product application have been provided.*

Reviewer #2:

However, I struggled with the breadth of the effort and indeed wonder whether is might be better represented if provided as two publications: one on modelling and another on repellents testing. Suggested as, at least for me, the modelling efforts lose some sharpness because of the repellents testing discussion and vice versa. To be clear, this is an observation for consideration and not a recommendation.

*Thank you. We did consider the benefits of presenting the work as two manuscripts, but saw value in combining both experimental work and a theoretical exploration of how the treated clothing could reduce cases of infections.*

General comments:

Text could/should be added to indicate that appropriate authorizations/ethics approvals were in place to support the research (or alternatively that these were not required). This point raised as, at least in North America, research authorizations/approvals often are required to test repellents with human subjects and/or for unlicensed/unregistered compounds.

*We have noted in the revised manuscript that no ethics committee approvals were needed. Approval would have been sought if we intended to recruit external participants.*

The cost effectiveness analyses (CEA) approach and related discussions could be improved/expanded (see below comments for more detail). For example, was discounting applied, were potential repellent harms considered (time to apply, health effects).

*Potential harms of repellent use were not considered in the model, as none are known or have been quantified. We have added this to the Methods (lines 251-252). Specific comments have been addressed below.*

Specific comments (by line number):

Lines 20-44 (Abstract/Author Summary).

- "...protected form infectious bites when outdoors". Such protection might also be warranted indoors esp. for important Aedes vectors such as Ae. aegypti.

*We agree, but our primary focus here is finding tools that could be used outside where people do not have the protection offered by window screens or other interventions.*

- I read the abstract before the rest of the text and wondered (aloud) about the range of values modelled for repellent performance, e.g., the values cited in the abstract are on the "best case" side of things with respect to user uptake. Of course, further reading highlighted that the authors generated estimates with a comprehensive range of values. The abstract would (IMO) be improved by including a better representation of the entirety of the range of model inputs considered and the generalities that emerge from this, i.e. the crucial importance of user uptake.

*There is not space within the word restriction to add a full range but we have given another example that we think is useful.*

- "...repellents can be considered as highly cost-effective"...add appropriate caveats/context. For example, something like "...our modelling work provides support to the concept that effective repellents, i.e. that are used regularly and appropriately, could be cost-effective interventions...".

*Thank you, we have used this wording in the revised manuscript.*

Lines 59-65: Discussion of vaccines should be tidied up. For example:

- YFV is a LAV and is reactogenic with the potential for (rarely) severe/life-threatening AE. Suggest avoiding the trigger words such as “safe” and rather indicate it is an effective, widely used and is recommended by the WHO, etc.

*This has been addressed.*

- Chik vaccine (IXCHIK) is authorized in the US and QDENGA is authorized but EMA. So, other vaccines are available (though admittedly not widely available/deployed).

*Thank you, the wording has been changed.*

Lines 66/67: PPT prevent bites, often by preventing contact, but not always. For example, it is not unusual for mosquitoes to contact (if only briefly) DEET protected skin.

*The wording has been modified.*

Lines 176 to 189. Perhaps move Tables 1 & 2 to supplemental materials?

*Thank you for the suggestion. We have left Tables 1 & 2 in the main manuscript, because we think that the analysis is easier to follow with the Tables in the main text.*

Line 203 to 235. CEA analysis.

- Was discounting rate (e.g., 3%?) applied to future costs/benefits?

*Discounting was not applied to short-term costs and benefits because we consider the potential impact of a repellent over a period of time that could be considered a single outbreak event, rather than over an extended timeframe over which discounting would become important. Value of lost labour or long-term sequelae of disease were extracted from the literature as total costs to which discounting had already been applied. A statement to explain this has been added to line 249 of the main manuscript.*

- Is the analyses perspective societal and/or health care system (vs individual)?

*As we consider the total cost of lost labour in addition to the total cost for healthcare over all infected individuals, the perspective is societal. We have clarified this in lines 227-228 in the main manuscript.*

- What was the CEA threshold, cost neutrality, i.e. no cost or better?

*We calculate the maximum cost per repellent application that is less than or equal to the value of the benefits from repellent application. So the CEA threshold is cost neutrality. We have added an explanation to lines 251-252 of the main manuscript.*

Lines 367-384.

- Was the assumption a closed population w/o immigartion/emigration, i.e. simulated “persons” were present for the entirety of the outbreak event? If yes, indicate such in text. Also, perhaps tough on its implication including, for example, that estimates would not be apt for persons who were not present for the entirety of an event such as travellers.

*The model considers only the population in CHKV/DNV/ZKV endemic regions, not travellers. Therefore, no in- or out-migration of people is included in the analysis. We have explained this in lines 197-199 of the Methods and added a caveat that CE thresholds derived from this analysis do not apply to travellers to the Discussion (lines 551-552).*

- Use terminology more representative of what was done and that makes clear that estimates were generated with a model and are prone to limitations thereof. Perhaps along the lines of "...in our model simulations, a repellent with 100% initial efficacy after application and a half-life of 7 hours (as found for 372 dUDL with our survival analysis) reduces the estimated burden of ZKV infection by 30% (outbreak R0=2.2)...".

*Line 398 has been changed as suggested.*

- Highlight the asymmetry of the benefits function, i.e. the estimated population benefit:population coverage function increases in a positive fashion? An important upshot is that repellent coverage must be high to achieve substantial impact under the scenario(s) evaluated with the model [sidebar: I do not believe high levels of population coverage are achievable with PPT such as repellents, something that is generally borne out in the literature].

*We have highlighted the asymmetric nature of the benefit-coverage function in the Results (lines 413-414). References for low achieved coverage in practice and high reported coverage in intervention trials have been added to the Discussion (lines 532-533).*

Lines 404-408.

- Use terminology more representative of what was done and that makes clear that estimates were generated with a model and are prone to limitations thereof. Perhaps along the lines of "...in our CEA simulations, a repellent with 80% initial efficacy and twice daily application was estimated to avert total health care costs per person of...".

*Lines 433-435 in the revised manuscript have been changed as suggested.*

Line 415 and Table 5. Consider adding text to indicate that the estimated cost per application to achieve the CEA threshold increases substantially (by > 20 x over the range of values considered) with decreasing population coverage?

*A sentence to highlight this observation has been added to lines 445-447 of the Results in the main manuscript.*

Lines 429 to 431. There is abundant literature to suggest low levels of compliance is often the case among travellers.

*Thank you. We have not found abundant literature but cited two sources show lower than ideal rates of compliance.*

Lines 500-511. Suggest a more fulsome discussion of whether current modalities, including dUDL, are likely to meet the target product profile for a PPT that would effectively reduce the burden of infection during an outbreak, i.e. high population coverage AND high efficacy.

*Thank you, we already have a very long discussion but have added this point and some study limitations.*
